# A First-in-Human Phase I Clinical Study with MVX-ONCO-1, a Personalized Active Immunotherapy, in Patients with Advanced Solid Tumors

**DOI:** 10.1158/2767-9764.CRC-24-0150

**Published:** 2024-08-14

**Authors:** Rémi Vernet, Eugenio Fernandez, Denis Migliorini, Virginie Ancrenaz, Emily Charrier, Marie-Claude Belkouch, Olivier Von Rohr, Muriel Urwyler, Claudio De Vito, Jessica Renaux, Jean Villard, Olivier Rubin, Julien Grogg, Nicolas Mach

**Affiliations:** 1 Department of Oncology, Geneva University Hospitals and Medical School, Geneva, Switzerland.; 2 Centre for Translational Research in Onco-Hematology, University of Geneva, Geneva, Switzerland.; 3 Agora Cancer Research Center, Lausanne, Switzerland.; 4 Swiss Cancer Center Léman (SCCL), Lausanne, Switzerland.; 5 Division of Clinical Pathology, Diagnostic Department, Geneva University Hospitals, Geneva, Switzerland.; 6 MaxiVAX SA, Geneva, Switzerland.; 7 Clinical Cell Therapy Lab, Geneva University Hospital, Geneva, Switzerland.

## Abstract

Over two decades, most cancer vaccines failed clinical development. Key factors may be the lack of efficient priming with tumor-specific antigens and strong immunostimulatory signals. MVX-ONCO-1, a personalized cell-based cancer immunotherapy, addresses these critical steps utilizing clinical-grade material to replicate a successful combination seen in experimental models: inactivated patient’s own tumor cells, providing the widest cancer-specific antigen repertoire and a standardized, sustained, local delivery over days of a potent adjuvant achieved by encapsulated cell technology. We conducted an open-label, single-arm, first-in-human phase I study with MVX-ONCO-1 in patients with advanced refractory solid cancer. MVX-ONCO-1 comprises irradiated autologous tumor cells coimplanted with two macrocapsules containing genetically engineered cells producing granulocyte–macrophage colony-stimulating factor. Patients received six immunizations over 9 weeks without maintenance therapy. Primary objectives were safety, tolerability, and feasibility, whereas secondary objectives focused on efficacy and immune monitoring. Data from 34 patients demonstrated safety and feasibility with minor issues. Adverse events included one serious adverse event possibly related to investigational medicinal product and two moderate-related adverse events. More than 50% of the patients with advanced and mainly nonimmunogenic tumors showed clinical benefits, including partial responses, stable diseases, and prolonged survival. In recurrent/metastatic head and neck squamous cell carcinoma, one patient achieved a partial response, whereas another survived for more than 7 years without anticancer therapy for over 5 years. MVX-ONCO-1 is safe, well tolerated, and beneficial across several tumor types. Ongoing phase IIa trials target patients with advanced recurrent/metastatic head and neck squamous cell carcinoma after initial systemic therapy.

**Significance:**

This first-in-human phase I study introduces a groundbreaking approach to personalized cancer immunotherapy, addressing limitations of traditional strategies. By combining autologous irradiated tumor cells as a source of patient-specific antigens and utilizing encapsulated cell technology for localized, sustained delivery of granulocyte–macrophage colony-stimulating factor as an adjuvant, the study shows a very good safety and feasibility profile. This innovative approach holds the promise of addressing tumor heterogeneity by taking advantage of each patient's antigenic repertoire.

## Introduction

Cancer vaccines have come a long way to be finally recognized as a promising modality to treat cancer. The efforts of the past decades were rewarded with modest success, and it was only the great advances in developing better methods and a deep understanding of the immune system and its interaction with cancer that supported the discovery and development of promising vaccine candidates (1, 2). Numerous preclinical and clinical studies have shed light on the two important elements of effective vaccination against cancer: (i) the target of (neo)antigen and (ii) the adjuvant to enhance antigenicity and stimulate an immune response (3). A broad spectrum of novel antigen-presenting strategies is under investigation, with a focus on tumor-associated antigens or tumor-specific antigens. Vaccine antigen strategies have lately focused on tumor-specific antigens, often called neoantigens, because they are deemed to trigger the initiation of a specific T-cell response. However, the downside of this approach is the differential expression of neoantigens in tumor cells. Neoantigens may be expressed as high-affinity antigens in some cells and may be entirely lost in others (4, 5). However, tumor vaccines targeting one or several specific tumor antigens cannot include all tumor-cell information relevant to immunity, such as whole-antigen repertoire that is required for immunity pattern recognition (6). Recently, a combination of mRNA-based neoepitope vaccine plus atezolizumab plus chemotherapy raised some interesting results in a phase I trial in the adjuvant setting in resected pancreatic adenocarcinoma (7). In the current study, we used a whole–tumor-cell preparation, inactivated by irradiation, aiming at presenting a very broad antigenic repertoire including any cancer-specific targets.

However, unmodified tumor-cell preparations usually trigger only minor immune response (8), clearly illustrating the need for potent adjuvants. Many immunostimulatory cytokines have been evaluated with tumor-cell vaccines, and granulocyte–macrophage colony-stimulating factor (GM-CSF) emerged as one of the most potent adjuvants in generating antitumor immunity (9, 10). One technology holding promise is GM-CSF–modified vaccines known as GVAX that have been extensively studied in humans (11, 12). Despite the excitement engendered by experimental animal models and early-phase human trials, GVAX has not lived up to its promise in inducing clinically meaningful outcomes in patients with cancer. Lack of prolonged, sustained, local delivery of GM-CSF by unprotected allogeneic cells is a likely explanation. Another cause may be related to the dual and sometimes opposing effects of GM-CSF in inducing the recruitment of regulatory T cells and myeloid-derived suppressor cells (13). Multiple studies have now demonstrated that GM-CSF can trigger a strong immunogenic response or instead lead to tolerogenicity when delivered at high dose and/or in a systemic way. To optimize the potent adjuvant effect of GM-CSF, we have designed and successfully tested encapsulated cell technology as a way to secure standardized, stable, and local prolonged release of GM-CSF at the immunization site. Encapsulated cell technology is an effective approach for the continuous and local delivery of therapeutic proteins released by genetically modified allogeneic cells.

Here, we report data from the first-in-human, phase I study of MVX-ONCO-1, a patient-specific cancer immunotherapy combining irradiated autologous tumor cells and encapsulated allogeneic cells producing GM-CSF. This open-label, single-arm study was designed to assess the safety, tolerability, and signals of the efficacy of MVX-ONCO-1 administered subcutaneously for the treatment of patients with advanced cancers progressing after standard therapies.

## Materials and Methods

### Study design and participants

This clinical trial is an open-label, single-center, phase I study in patients with advanced metastatic tumors. Thirty-four patients were treated at Geneva University Hospitals between May 5, 2014, and November 24, 2021. Patients’ characteristics are listed in Table 1. Eligible patients were ≥18 years old with advanced metastatic solid cancer in progression in which all standard treatments were exhausted or not feasible, with an estimated life expectancy of at least 4 months, Eastern Cooperative Oncology Group performance status grade 0 to 2, and no major impairment of liver, renal, and hematologic functions. Eligible patients also had to present with a primary tumor and/or metastasis amenable for partial/total surgery or tap and subsequent cell harvesting estimate >27 × 10^6^ cells. Patients were excluded if they had participated in any other investigational study or received an experimental therapeutic procedure or chemotherapy treatment within 4 weeks of screening, if they were suffering from a systemic disease not controlled by usual medication, if they presented with untreated brain metastasis, if they were on chronic immunosuppressive treatment or therapeutic anticoagulation with coumarin or continuous intravenous heparin, if they had tested positive for human immunodeficiency virus 1 and 2, human T-cell leukemia–lymphoma virus 1, hepatitis B surface antigen, or hepatitis C antibody, if they were females of child-bearing potential who were pregnant or lactating or who were not using adequate contraception, or if they presented with a known allergy to reagents in the study product like penicillin or streptomycin. All prospective participants received written and verbal information about the study at a prior interview and signed informed consent prior to any study-specific procedure.

**Table 1:** Patient demographics and baseline characteristics

Parameter	Safety population
Number of patients	34
Age (years)	
Minimum–maximum	32–89
Mean (SD)	57.9 (14.21)
Median	55.5
Ethnic group	*n* (%)
Caucasian	32 (94.1)
Asian	1 (2.9)
Other	1 (2.9)
Sex	*n* (%)
Male	15 (44.1)
Female	19 (55.9)
ECOG status	*n* (%)
0	15 (44.1)
1	13 (38.2)
2	6 (17.6)
Previous lines of systemic therapy	
0–1	6 (17.6)
2–4	18 (52.9)
≥6	10 (29.4)
Tumor type	*n* (%)
Chordoma	11 (32.4)
Colon carcinoma	3 (8.8)
Gastric carcinoma	1 (2.9)
HNSCC	2 (5.9)
Melanoma	1 (2.9)
Ovarian carcinoma	8 (23.5)
Pancreatic carcinoma	3 (8.8)
Peritoneal carcinoma	1 (2.9)
Prostate mesothelioma	1 (2.9)
Saliva gland cancer	1 (2.9)
Soft tissue sarcoma	2 (5.9)

Abbreviation: ECOG, Eastern Cooperative Oncology Group.

### Procedure

The entire manufacturing process adhered to Good Manufacturing Practices within a Swissmedic-certified cell therapy facility. The MVX-ONCO-1 treatment was administered at the Oncology Department of Geneva University Hospitals by qualified physicians following Good Clinical Practices and consists of the following two components:

1 Patients’ autologous tumor cells, harvested from either the primary tumor or a metastasis, by a surgical procedure or an aseptic tap from malignant ascites or pleural fluid. Tumor cells are processed into a single-cell suspension through physical (gentleMACS Octo Dissociator) and enzymatical digestion (Collagenase NB6). A minimum of 27 × 10^6^ cells harvested are required for enrollment [six treatments + three delayed-type hypersensitivity (DTH) tests]. After 100 Gy irradiation, aliquots of 4 × 10^6^ cells are prepared and stored frozen in liquid nitrogen. Each dose is prepared as a single-cell suspension and resuspended into 0.5 mL Hank’s Balanced Salt Solution + calcium + magnesium for subcutaneous injection.
2 Two biocompatible polyethylsulfone macrocapsules, containing MVX-1 cells (8 × 10^5^ cells in each macrocapsule), placed underneath the skin away from any tumor deposit. Macrocapsules are composed of materials broadly used in medicine and well described in the literature (14, 15). MVX-1 is the MaxiVAX-1–certified cell line for human use in clinical trials. These cells are K562 cells genetically modified to secrete human GM-CSF. Macrocapsules are loaded with MVX-1 cells and then sealed with UV-sensitive biocompatible glue for subcutaneous implantation. Similar K562 cells engineered to produce GM-CSF have already been used in several clinical studies (16, 17). The macrocapsule can be maintained in culture under controlled conditions (5% CO_2_ and 37°C) for 1 month, ensuring stable GM-CSF secretion.

**Figure 1:**
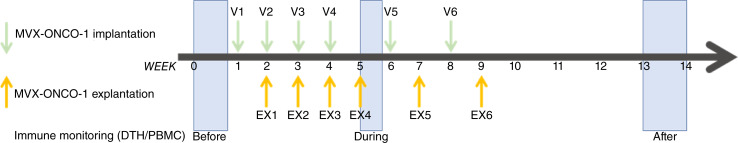
Treatment and immune monitoring schema.

Each MVX-ONCO-1 treatment consists of subcutaneous implantation, 1 cm apart in a parallel manner, of two macrocapsules containing GM-CSF–secreting cells and subcutaneous injection of lethally irradiated autologous tumor cells between the two macrocapsules. MVX-ONCO-1 is administered weekly for 4 weeks (weeks 1, 2, 3, and 4), followed by two additional immunization 2 weeks apart (weeks 6 and 8). Macrocapsules are removed 1 week postimplantation (Fig. 1).

### Endpoints and assessments

The primary objective of this study was to assess the feasibility of the subcutaneous implantation of both macrocapsules and tumor-cell suspension as well as evaluating the safety and tolerability of the treatment. Collecting signals of efficacy and immune education were secondary objectives of this phase 1 trial.

Safety assessment was evaluated by clinical assessments, vital signs, local and systemic tolerance, laboratory tests, and electrocardiograms and were conducted from baseline until week 18. Patients could be discontinued from study treatment for unacceptable toxicity, pregnancy, or patient decision. Adverse events (AE) were graded using Common Terminology Criteria for Adverse Events v.4.0 and reported using Medical Dictionary for Regulatory Activities v.25.0 until week 18, whereas related AEs and serious adverse events (SAE) were recorded until the end of participation in the study. Patients were then followed up for survival status and SAE until death or for 5 years, whichever came first.

Efficacy was assessed by follow-up of the patient’s survival at 6, 12, and 18 months, and disease status using RECIST 1.1 at baseline and then at weeks 6, 12, and 18 with a cutoff date as of April 16, 2022. Additional information on tumor status beyond week 18 was obtained for specific subject after signed agreement.

### DTH

DTH tests were performed with intradermal injections of 1 × 10^6^ irradiated autologous tumor cells in healthy skin before, during, and after treatment (Fig. 1). Erythema, induration, and ulceration at the sites of injections were measured to determine positivity of the test, and a punch biopsy of the injection site was collected to analyze recruited immune cells.

### *Ex vivo* IFNγ enzyme-linked immunospot

Peripheral blood mononuclear cells (PBMC) were harvested and frozen before, during, and after treatment. For the *ex vivo* IFNγ enzyme-linked immunospot (ELISpot) experiment, the cells were thawed in RPMI 1640 medium (Thermo Fisher Scientific, 72400021) containing 10% heat-inactivated FBS (Thermo Fisher Scientific, 10101-145) and 1% penicillin–streptomycin (Thermo Fisher Scientific, 15140122) and rested overnight under a controlled atmosphere (5% CO_2_ and 37°C). Subsequently, the ELISpot assay was performed with PBMCs coincubated with freshly thawed autologous irradiated tumor cells at a ratio of 1:1 (10^5^ cells of each) or with purified Brachyury protein (1 µg/mL; Acris) per well for 18 to 24 hours using precoated 96-well plates (Mabtech). PBMCs alone and tumor cells alone were used as negative controls. All conditions were run in triplicate and cultured in serum-free X-VIVO 15 medium (Lonza, BE02-060F). The plates were washed according to the manufacturer’s instructions and counted using the iSpot Robot ELISpot reader (Autoimmun Diagnostika GmbH). The final number of spot-forming units was calculated after background subtraction (negative controls).

### Statistical methods

The feasibility analysis set included all patients who had enough tumor cell harvested. The safety analysis set included all participants who received MVX-ONCO-1 at least once. The efficacy analysis set included all participants who received MVX-ONCO-1 at least once and who had at least the week 6 post-baseline efficacy measurement. The descriptive analysis of the safety and efficacy results was performed once the last enrolled patient had completed the week 18 assessments. Statistical analyses were performed using SAS version 9.4.

### Data availability

Data are available on reasonable request. The data of this study have been deposited in a research database. All requests should be submitted to the corresponding authors.

## Results

### Feasibility

**Figure 2:**
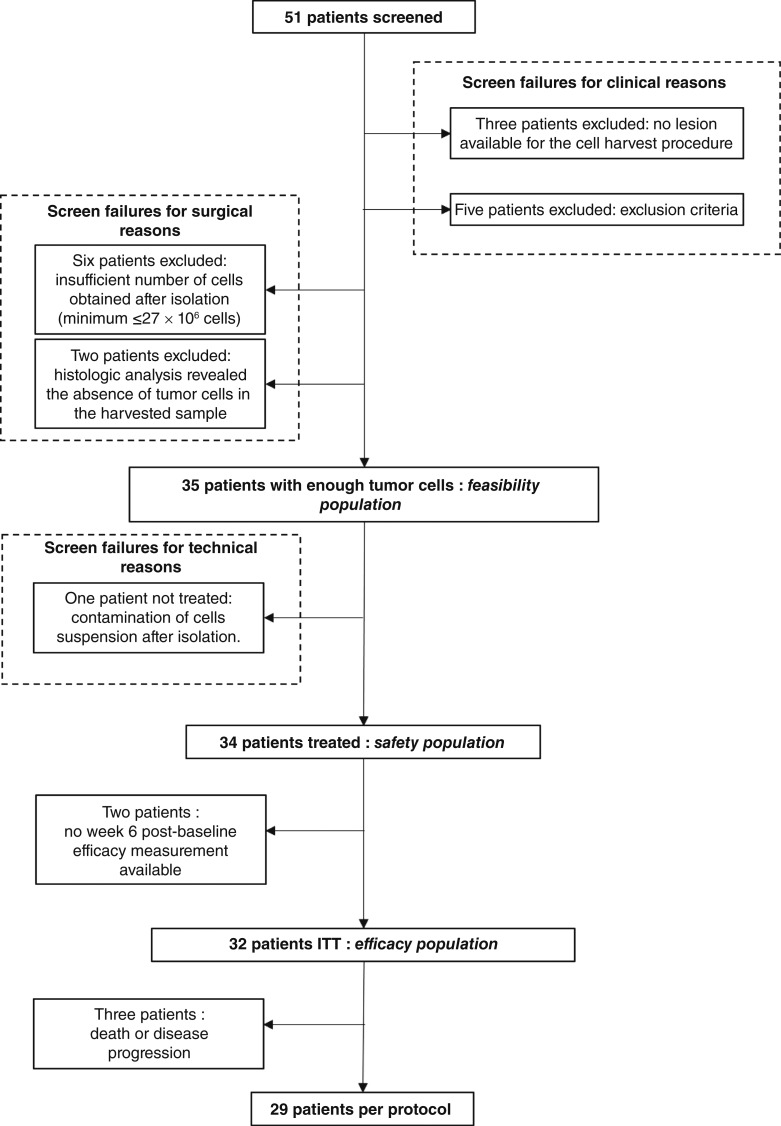
Trial profile. A screened population included all patients who were screened. A safety population comprised 34 treated patients who received at least one dose of the study treatment. The ITT set comprised all patients who had received at least one dose (defined as at least 1-day application without removal of the macrocapsules) of the study treatment and for whom a week 6 post-baseline efficacy measurement was available.

Enrollment for the study was completed by December 2021. A total of 51 patients were screened initially (Fig. 2). However, of these 51 screened patients, 16 patients were excluded from the study because of clinical and surgical screen failures. These exclusions could be related to various factors, such as medical conditions or surgical complication risk, which made these patients ineligible for participation in the study. As a result, the study included 35 patients who fulfilled the selection criteria, signed the consent forms, and had sufficient tumor cells harvested to prepare the investigational medical product. One patient, who responded to MVX-ONCO-1 [partial response (PR)] with prolonged clinical benefit, was re-enrolled because of a late relapse more than 2 years after first treatment and is depicted as two patients: “MVX-01-21” then “MVX-01-38”.

In terms of feasibility, it is mentioned that only 1 patient of the 35 patients (2.9%) was not treated. This particular patient could not receive the treatment because of an investigational medical product defect. Specifically, the suspension of irradiated autologous tumor cells, which is a part of the investigational medical product, was found to be contaminated. As a precautionary measure, this patient was not administered the treatment. With regard to the manufacturing of macrocapsules under Good Manufacturing Practices guidelines, no feasibility issues were encountered. According to the protocol, a 2-week safety window applies between autologous tumor cell harvesting and the first vaccination. This window allows to check for any out of specification results. In this trial, time from harvesting tumor cells to first treatment was less than 3 weeks. All manufactured batches met our release criteria, which include a minimum secretion per capsule of 20 ng GM-CSF per day, 6 to 7 days after loading of the MVX-1 cells into macrocapsules without any bacteriologic contamination. On the day of treatment, the time required for dosing patients, from the thawing step of the irradiated autologous tumor cells to preparing the macrocapsules and implanting them in the patient, was consistently within 4 hours (±2 hours) for all patients. Following preparation, the determined expiration timeline for the product was established to be 12 hours under controlled temperature (4°C for the irradiated autologous tumor cells and 37°C for the macrocapsules).

A total of 34 treated patients were included in the study. The demographic and baseline characteristics of these 34 patients are depicted in Table 1. Among the treated patients, the majority (91.2%) had tumor not considered prone to immunotherapy (cold tumors), whereas only three patients, two head and neck squamous cell carcinoma (HNSCC) cases and one melanoma case, had potentially immune-responsive cancers (hot tumors). This indicates that the cold tumor type was the most prevalent among the patients included in this study. Moreover, 82.3% of patients had received more than two previous lines of therapies: more than half of patients (52.9%) had received between two and four previous lines of therapies and 29.4% of patients had received more than five previous lines of therapy, reflecting the advanced stages and the aggressiveness of their diseases.

### Safety

All 34 patients received MVX-ONCO-1, with each patient receiving at least one dose of the treatment. The safety assessment was conducted on the entire group of 34 patients. This means that both safety profile and potential AEs of the treatment were evaluated in all patients who received MVX-ONCO-1. Of the 34 treated patients, 29 (85.3%) completed the full six administrations of MVX-ONCO-1, whereas 2 patients did not have week 6 post-baseline efficacy measurement, and 3 patients did not complete the treatment because of death or disease progression (Fig. 2).

AEs were observed in all 34 patients participating in the study, primarily attributed to disease progression and associated symptoms. However, the most commonly reported AE (32.4%) was implant site hematoma, which did not have any significant impact on the patient’s life-threatening condition or treatment regimen. Fatigue was the second most frequently observed AE (29.4%). Table 2 presents the most commonly observed AEs, reported in three or more patients. The severity of AEs varied, with four patients (11.8%) experiencing life-threatening events or 15 patients (44.1%) experiencing severe events, all related to disease progression. No patient withdrew from the study because of adverse events.

**Table 2:** Summary of most common AEs (in three or more patients)

AE	Number (%) of patients
Implant site hematoma	11 (32.4)
Fatigue	10 (29.4)
Abdominal pain	6 (17.6)
General physical health deterioration	5 (14.7)
Disease progression	5 (14.7)
Ascites	4 (11.8)
Constipation	4 (11.8)
Nausea	4 (11.8)
Dyspepsia	4 (11.8)
Implant site hemorrhage	3 (8.8)
Implant site pain	3 (8.8)
Pyrexia	3 (8.8)
Arthralgia	3 (8.8)
Dyspnea	3 (8.8)
Myalgia	3 (8.8)
Anemia	3 (8.8)

In this heavily pretreated frail population, mortality rates showed that 14 patients (41.2%) died during the initial 18 weeks, and as of the data cutoff date, additional 13 patients (38.2%) had died after week 18, with none of the deaths being attributed to the study treatment.

SAEs were reported in 30 patients, with only 1 event (neck pain) having a possible connection to the treatment. Additionally, laboratory abnormalities directly linked to disease progression were reported as AEs in 12 patients, none considered related to the study treatment. One patient had two episodes of supraventricular tachycardia.

Finally, a total of 384 macrocapsules were implanted in 34 patients. Of all these implanted macrocapsules, 17 (4.4%) macrocapsules were slightly bent at removal, which had no impact on the treatment or on the patient’s health. In four cases (1%), the macrocapsule had a broken suture string, requiring a small incision to remove the macrocapsule. Furthermore, in one case (0.3%), a macrocapsule was damaged during removal. Two additional cases (0.5%) were not further described by the physician.

### Efficacy

For the efficacy analysis, the analysis was performed on the intent-to-treat (ITT) population. The ITT population consisted of 32 patients. The ITT analysis includes all patients who were initially assigned to receive the treatment, regardless of whether they completed the treatment or withdrew from the study prematurely and for whom a week 6 post-baseline efficacy measurement was available (Fig. 2).

### Antitumor activity

**Figure 3:**
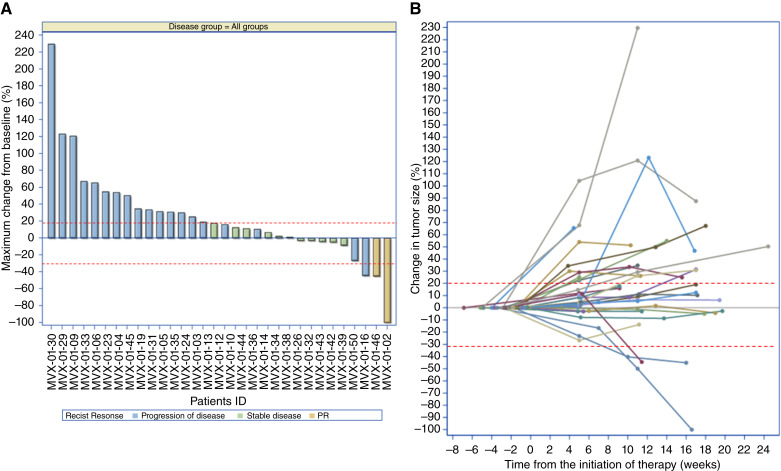
Antitumor activity of MVX-ONCO-1. **A,** Waterfall plot of the maximum percentage of tumor size change in each patient. **B,** Percentage changes from baseline of tumor size (sum of longitudinal change of recorded lesions) over time in each patient.

The efficacy analysis of tumor response demonstrated the following results (Fig. 3): Two patients [one recurrent/metastatic HNSCC (R/M HNSCC) “MVX-01-02” and one chordoma “MVX-01-21”] of 32 experienced a PR after 12 weeks of MVX-ONCO-1 treatment (Fig. 3A) and then maintained the PR at week 18, indicating a significant reduction of 30% in tumor size compared with the baseline (Fig. 3B). The disease was stable in 18 patients (56.3%) at week 6, 10 patients (31.3%) at week 12, and 6 patients (18.8%) at week 18, indicating that their tumor did not show significant growth or shrinkage during those time points (Table 3). Disease progression was observed in 14 patients (43.8%) at both weeks 6 and 12 and in 8 patients (25.0%) at week 18, indicating an increase in tumor size and/or new metastatic lesions (Table 3). The best overall response, taking all time points into account, was a PR in two patients (6.3%). The disease control rate, which combines complete response (CR), PR, and stable disease, was 56.3% (18 of 32 patients), indicating that a significant proportion of patients showed either a reduction in tumor size or stable disease during the study period. PR, prolonged survival combined with evidence of tumor-specific immune response, was observed in a relapsing chordoma patient, as published in a case report previously (Table 3; ref. 18). In the long-term follow-up, we also observed a complete remission upon palliative chemotherapy in one of the two patients with R/M HNSCC. This patient, heavily pretreated with radiotherapy, cisplatin, docetaxel, carboplatin, 5-fluorouracil, and pembrolizumab with progressive disease before enrolling in the study, had a progressive disease upon MVX-ONCO-1 treatment (>20% increased of target lesion with no new lesion) and a subsequent CR to a palliative regimen combining carboplatin and cetuximab. At the latest visit on December 2023, this patient remains in CR for more than 7 years, with no antitumor therapy for more than 5 years.

**Table 3:** Tumor responses according to RECIST

Response	Week 6	Week 12	Week 18	Best overall response, *N* (%)
*N* (%)	32 (100)	27 (84.4)	17 (53.1)	32 (100)
PR	0	2 (6.3)	2 (6.3)	2 (6.3)
Stable disease	18 (56.3)	10 (31.3)	6 (18.8)	16 (50.0)
Progressive disease	14 (43.8)	14 (43.8)	8 (25.0)	14 (43.8)
Not evaluable/missing	0	1 (3.1)	1 (3.1)	–
Disease control rate	18 (56.3)			

### Survival

**Figure 4:**
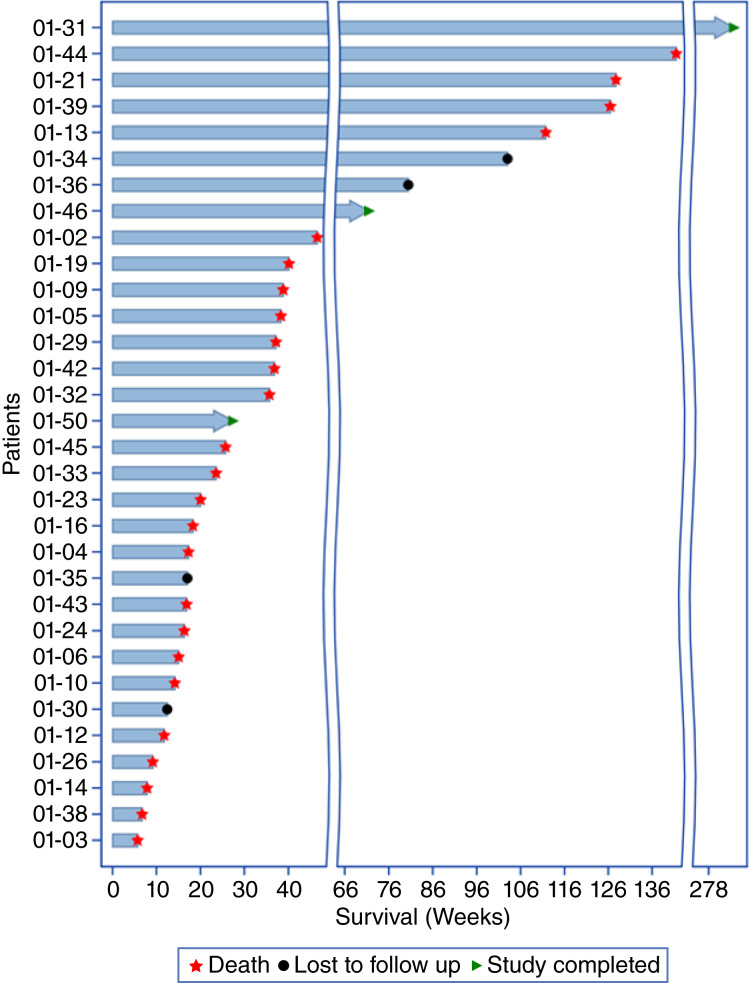
Swimmer plot showing OS of each patient since the start of MVX-ONCO-1 treatment. *, Number of patients who have theoretically reached the time point of interest at the cutoff date. Patients lost to follow-up are included.

The study observed signs of prolonged survival, as depicted in Fig. 4A and Table 4. Four patients were lost to follow-up, and the dates of their last contact were included in the analysis. The global median overall survival (OS) was determined to be 186 days (Table 5). Specifically, 53% of patients were alive 6 months after their initial treatment with MVX-ONCO-1. The survival rates at 12 and 18 months were 25.8% and 23.3%, respectively. Additionally, 20% of patients were still alive 24 months after starting the treatment (Fig. 4A).

**Table 4:** Summary of OS of each patient since the start of MVX-ONCO-1 treatment

	Total *N*^a^	*N* (%) alive	*N* (%) lost to follow-up
Status at 6 months	32	17 (53.1)	2 (6.3)
Status at 12 months	31	8 (25.8)	2 (6.5)
Status at 18 months	30	7 (23.3)	2 (6.7)
Status at 24 months	30	6 (20)	3 (10)

a Number of patients who have theoretically reached the time point of interest at the cutoff date. Patients lost to follow-up are included.

**Table 5:** OS (days) per tumor response and per tumor type

Tumor Response	Median OS (IQR)
PR	610 (284)
Stable disease	254 (484)
Progressive disease	125 (136)
Tumor type (*n*)	Median OS (IQR)
All (32)	186 (258)
Chordoma (11)	258 (661)
Ovarian carcinoma (8)	125 (147)
Pancreatic carcinoma (3)	99 (95)
Colon carcinoma (3)	105 (223)
Tumor type (*n*)	Mean OS (IQR)
HNSCC (2)	1156 (830)
Tumor type (*n*)	OS (IQR)
Soft tissue sarcoma (1)	260 (NC)
Prostate carcinoma (1)	140 (NC)
Melanoma (1)	180 (NC)
Peritoneal mesothelioma (1)	118 (NC)
Salivary gland cancer (1)	782 (NC)

Abbreviation: NC, non calculated.

As our patient cohort is heterogeneous in cancer type, the median OS, the mean OS, or the OS depending on the number of patients for each type of cancer were also analyzed separately, and this information can be found in Table 5. Among all the different tumor types in our study population, two patients with HNSCC stand out with a remarkable mean OS of 1,156 days, surpassing the survival outcomes of all other tumor types.

### Immune response

**Figure 5:**
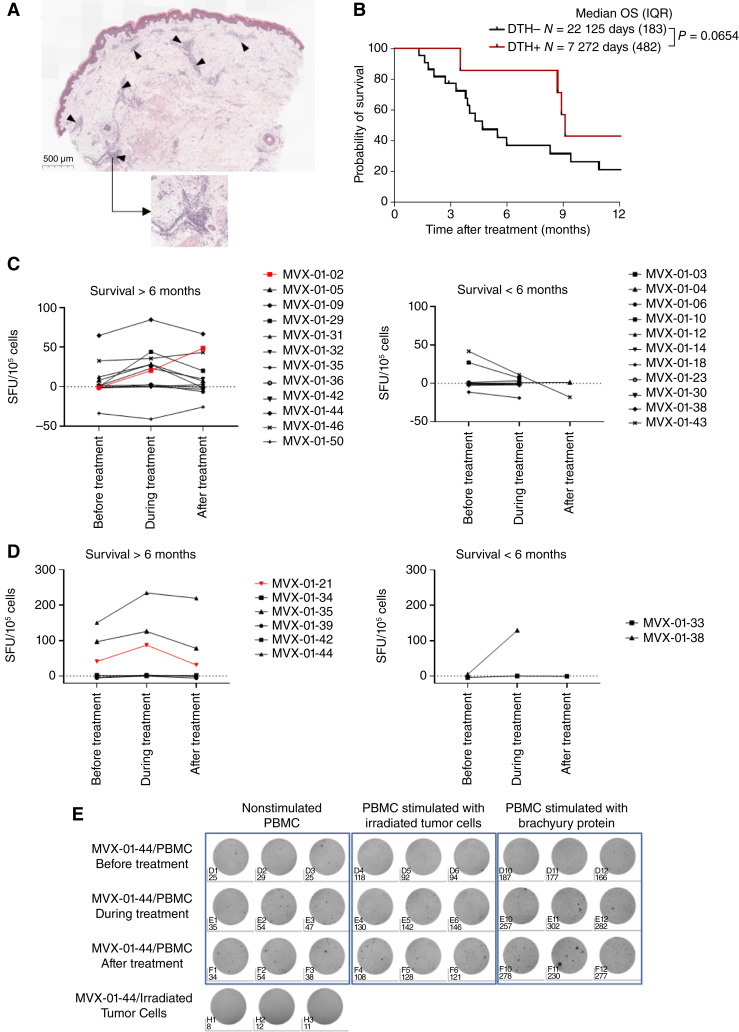
*In vivo* and *ex vivo* immune responses against autologous irradiated tumor cells or tumor-associated antigens. **A,** Hematoxylin and eosin staining of a skin punch biopsy of the DTH site showing a representative perivascular area of immune infiltration (black arrow). **B,** Kaplan–Meyer survival curve and median OS according to DTH status. **C** and **D,** IFNγ ELISpot of PBMC secretion after stimulation. Total PBMCs (105 per well) from patients at three different time points (before, during, and after treatment) were incubated in the presence of autologous irradiated tumor cells (**C**) or Brachyury protein (**D**). Representative plate images for MVX-01-44 PBMC, nonstimulated (left), stimulated with irradiated tumor cells (middle), or with Brachyury protein (right) is depicted in **E**. The final number of spot-forming units (SFU) was calculated after background subtraction (negative controls). All conditions were run in triplicate. Patient with PR are highlighted in red.

Qualitative assessment of immune-mediated response by DTH with irradiated autologous tumor cells were performed before, during, and after MVX-ONCO-1 treatment. After 48 to 72 hours, erythema, induration, and ulceration at the sites of injections were measured. The test was positive if the largest diameter measured was ≥5 mm. A skin punch biopsy was performed at the site of injection. The local reaction observed was associated with a strong perivascular inflammation in the punch skin biopsy of the DTH site (Fig. 5A). Patients were then classified according their DTH response status. If a patient acquired a positive reaction during and/or after MVX-ONCO-1 treatment, they were classified as positive. If a patient exhibited a negative DTH reaction during and after MVX-ONCO-1 treatment, they were classified as DTH negative. Twenty-two patients (64.7%) were DTH negative, whereas seven (20.6%) were DTH positive. Last patients were classified as inconclusive (3, 8.8%) or deleterious (2, 5.9%) DTH responses due to missing information or change of positive to negative response, respectively. Interestingly, when positive, DTH was associated with a longer OS, as depicted in Fig. 5B.

Moreover, a quantitative assessment of immune response by the IFNγ ELISpot assay using irradiated autologous tumor cells or tumor-associated antigen to stimulate PBMCs was performed. Fifty percent of patients who had survived beyond 6 months showed an increase in IFNγ spots during treatment compared with baseline (Fig. 5C and E), meaning that treatment with MVX-ONCO-1 can trigger a tumor-specific immune response from PBMCs in this patient population. In contrary, all patients with survival status of less than 6 months did not mount any specific immune responses. Moreover, in patients diagnosed with chordoma and harboring Brachyury-positive tumor cells, PBMCs were also stimulated using Brachyury protein/peptides. Brachyury has been identified as a marker of chordoma cells, suggesting its potential role as an immune response initiator. (Fig. 5D and E). Among patients with a survival period exceeding 6 months, 50% exhibited a reactivity of their PBMCs against the Brachyury protein. This suggests that the immune response in individuals surviving beyond 6 months may be influenced by unidentified antigens. It is worth noting that patients demonstrating a PR are not necessarily those with the highest ELISpot responses. This suggests that the response to treatment is not solely influenced by T-cell response but can also be supported by other immune responses, such as humoral response.

## Discussion

Significant breakthroughs in the biological understanding of the immune system have led in the past years to the development of therapeutics such as, antibody–drug conjugates, immune checkpoint inhibitors (ICI), chimeric antibody receptor T cells, and RNA vaccines (7, 19–27). However, therapeutic cancer vaccines have not achieved significant success despite decades of research. Analyzing preclinical cancer models and past clinical vaccine development can help understand the factors involved in efficient anticancer vaccination and guide the crafting of a clinically meaningful approach recapitulating these required features. Optimal priming conditions are crucial to set the immune system for an efficient effector phase in which antigen-specific T cells and antibodies can efficiently recognize and destroy tumor cells. Creating this favorable niche for optimal antigen presentation and subsequent processing by antigen-presenting cells requires a very finely tuned setting. This goal is achieved by a sustained, controlled, low dose of GM-CSF at the subcutaneous vaccination site. In fact, preclinical studies have shown that subcutaneous injections of GM-CSF at the vaccination site can significantly increase the infiltration of dendritic cells (DC) in regional lymph nodes that drain the site of vaccination (28, 29). Genetically engineering tumor cells to secrete biologically active GM-CSF have also shown success in generating specific long-lasting protective systemic antitumor immune responses in preclinical studies. Such antitumor immunity has been demonstrated in multiple tumor types such as melanoma (30), bladder cancer (31), head and neck carcinoma (32), lymphoma (33), lung cancer (34), glioma (35), prostate carcinoma (36), neuroblastoma (37), acute myeloid leukemia (38), and breast cancer (39).

However, recombinant GM-CSF administered subcutaneously has not consistently proven effectiveness. The delivery method is critical, as low-dose, localized administration of GM-CSF display a potent adjuvant effect, whereas high doses can lead to immunosuppressive effects through the recruitment of tolerogenic DCs and myeloid-derived suppressor cells (40–43) and may explain many negative trials. To address these challenges, a biocompatible macrocapsule has been developed for subcutaneous implantation. It contains a proprietary immortalized cell line that continuously produces stable concentrations of GM-CSF, providing standardized delivery of a low-dose adjuvant over a week. In comparison with other trials in which daily high doses ranging from hundreds of micrograms to milligrams of GM-CSF are utilized, the average amount of secreted GM-CSF per macrocapsule in our study was below 250 ng and remained very low. This indicates that the delivered quantity of GM-CSF by our macrocapsules in our study is 1,000 times less than that used in other studies. Moreover, this macrocapsule acts as a physical barrier, preventing the encapsulated cells from being recognized and destroyed by the patient’s immune system. Although cell debris and/or subcellular components from the encapsulated cells may be found outside the macrocapsule and could potentially be processed by antigen-presenting cells to stimulate patient immunity, the encapsulated cells themselves will remain shielded from the patient’s immune reaction, ensuring the continued functionality of our encapsulated cell line. Additionally, MVX-1 cells lack HLA expression, making it highly improbable for enrolled patients to develop an immune response against MVX-1 cells.

Another important factor is selecting the antigenic target. Despite the characterization of various tumor-associated or -specific antigens, clinical trials have failed to demonstrate significant clinical benefits across tumor types. Recent interest lies in identifying and synthesizing patient-specific tumor neoantigens, particularly using mRNA liposomal formulations. Although single-agent activity has been limited, combining neoantigens with ICI has shown promise in patients with melanoma. However, immune evasion of neoantigens has been observed in lung and colorectal cancer studies, likely due to ongoing immune editing processes (44, 45).

In summary, understanding the factors involved in efficient anticancer vaccination, such as optimal priming conditions, precise delivery of GM-CSF, and selecting appropriate antigenic targets, is crucial for success. These insights have the potential to revolutionize personalized immunotherapies and improve outcomes for patients with various cancers.

The first generation of MVX-ONCO-1 takes a unique approach by using irradiated autologous tumor cells as the antigenic repertoire instead of selecting specific tumor antigens. This allows for quick processing and potential exposure to hundreds of tumor-specific targets without a selection process. In combination with two macrocapsules delivering GM-CSF, MVX-ONCO-1 aims to recapitulate the key features required in preclinical models. This approach has been evaluated in a first-in-human phase I clinical trial involving subjects with advanced metastatic progressive refractory tumors.

The study demonstrated that MVX-ONCO-1 has a very safe and well-tolerated profile. It can be administered in repeated doses without any evidence of a drug effect on vital signs, blood chemistry, hematology, or urinalysis evaluations. Notably, no SAEs related to the study drug were reported, and there were no clinically significant local or systemic reactions observed. Local inflammatory reactions could occur at the site of macrocapsule implantation such as swelling, itching, redness, or pain and are directly linked with the biological activity of the GM-CSF. The risk associated with subcutaneous implantation of macrocapsules containing a cell line producing human GM-CSF is estimated to be low. Additionally, no SAEs have been reported specifically related to the use of lethally irradiated autologous tumor cells in the context of antitumor immunization. This further supports the safety profile of MVX-ONCO-1 and suggests that this approach is well tolerated by patients.

Remarkably, in more than 50% of the treated patients, notable clinical effects were observed with MVX-ONCO-1. These effects ranged from the disappearance of lung metastasis to PR, prolonged disease stability, and unusually prolonged survival. It is worth noting that among the 32 patients, only 3 had cancers considered immunogenic (two head and neck carcinoma cases and one melanoma case), whereas the others fell into the category for which classical immunotherapies such as ICI have not been successful. Some of our patients have been exposed to ICI either before or after receiving MVX-ONCO-1. Although the numbers are very small, we have noted anecdotal but very impressive clinical observation for patients being exposed sequentially to both ICI and MVX-ONCO-1. In these heavily treated patients, it remains very difficult to attribute which line of treatment and/or which treatment sequence can be attributed to the tumor regression and contributed to patient survival. Based on these previous observations, only hypothesis can be formulated.

Previous studies by Hodi and colleagues (46) have also reported clinical benefits from subsequent ICI treatment in patients who received GM-CSF–secreting autologous tumor cells. Strong synergistic effects have been observed when combining GM-CSF immunization with anti-PD1 or CTLA4 blockade in various tumor models (47–50). None of these previous studies utilized MVX-ONCO-1 technology. The rationale for combining MVX-ONCO-1 with ICI is based on its potential to enhance the activation and recruitment of DCs, promoting a more robust antigen presentation. Additionally, the inclusion of ICI further contributes by unleashing the brakes on immune responses, allowing for a maximal sustained antitumor activity and overcoming inhibitory signals within the tumor microenvironment. This cooperative strategy aims to maximize the therapeutic impact on tumor immunity, potentially leading to better clinical outcomes. However, this potential synergy is still a hypothesis that has not been tested in clinical settings and requires validation through a comparative study.

Interestingly, the two patients with R/M HNSCC progressing after standard therapy developed immune response after MVX-ONCO-I treatment. In addition, we observed prolonged OS in these two patients including a very unusual clinical evolution with CR upon palliative therapy. This last patient is alive 7 years after enrollment in the study and in persistent complete remission with no anticancer therapy for more than 5 years. Based on these very interesting clinical findings, a multicenter efficacy phase IIa study in R/M HNSCC has recently been completed.

Overall, MVX-ONCO-1 represents an innovative, promising approach in cancer immunotherapy, combining irradiated autologous tumor cells as the antigenic repertoire and cell encapsulation technology for the controlled delivery of adjuvant. In this phase I clinical trial, MVX-ONCO-1 demonstrated a very safe profile and showed clinical effects in more than 50% of patients with advanced metastatic tumors, including tumor regression and prolonged survival. A phase IIa study, SAKK11-16, evaluating OS in metastatic HNSCC, aims to build upon these promising findings. MVX-ONCO-1 holds the potential to redefine personalized immunotherapies and improve outcomes for patients with cancer.

### Limitation of the study

The study design permits tumor assessment documentation only until week 18. Consequently, it is not possible to interpret the influence of other treatments after MVX-ONCO-1 treatment on patient survival. However, to provide readers with crucial clinical and scientific insights that may be relevant to the biological mechanisms of this innovative therapy, we obtained an additional informed consent from one study subject who has been in complete remission without any anticancer therapy for more than 5 years. This signed informed consent allows us to include information on his tumor kinetics beyond week 18 in this article.

## Supplementary Material

Table S1 Representativeness of Study Participants
